# Isolated Ear Clicks with Partial Voluntary Control

**DOI:** 10.5334/tohm.574

**Published:** 2020-12-16

**Authors:** François Voruz, Nils Guinand, Anthony E. Lang, Julien F. Bally

**Affiliations:** 1 Division of Otorhinolaryngology and Head and Neck Surgery, University of Geneva and University Hospitals of Geneva, Geneva, CH; 2 Edmond J. Safra Program in Parkinson’s disease and Morton and Gloria Shulman Movement Disorders Clinic, Toronto Western Hospital, Toronto, CA; 3 Division of Neurology, University of Geneva and University Hospitals of Geneva, Geneva, CH; 4 Service of Neurology, Department of Clinical Neurosciences, Lausanne University Hospital and University of Lausanne, Lausanne, CH

**Keywords:** palatal myoclonus, palatal tremor, tinnitus, ear click, functional, psychogenic

## Abstract

Background: Ear click is a rare type of objective tinnitus, classically described with associated palatal tremor/myoclonus (PT). Case report: A 15-year-old boy reported a constant bilateral ear clicking for 4 years, that could be stopped at will for a few seconds. Clinically, the ear clicks were audible without visible eardrum or palatal movement, and could be entrained by the examiner. Brain MRI was normal. Discussion: We propose to classify this as isolated ear clicks with partial voluntary control, putting it into context with other subcategories of “essential” or “isolated” PT.

## Introduction

One form of objective tinnitus (audible to an external observer) is referred to as an “ear click”. Ear click can be due to tremor or myoclonus involving the muscles attached to or surrounding the Eustachian tube (referred to as “peritubal” muscles), in which case it is classically described with associated palatal movement. We describe a case of objective clicking tinnitus without palatal tremor/myoclonus (PT) in which the ear clicks had semi-volitional characteristics.

## Case Report

A healthy 15-year-old boy reported a 4-year history of persistent spontaneous tinnitus in both ears. The tinnitus consisted of constant “clicks” at an irregular frequency, heard on both sides, independent of heart rate, present throughout the day but not during sleep. Clicks were not associated with other facial, cranial or body movements. There was no history of head trauma. No specific psychological trigger was identified. Neither the patient nor his family had any history of tics. The patient denied any urge or relief produced by the movements, and there was no waxing or waning. The patient reported the ability to willingly stop the tinnitus for a few seconds, especially by performing Valsalva maneuver. He denied any hearing or balance problem and had no history of neurological or otological disorder.

On clinical exam, audible irregular “clicks” were perceived on both sides of the patient’s head. Otoscopy revealed normal tympanic membranes without any visible movement (Video). Posterior rhinoscopy confirmed a normal anatomy of the Eustachian tubes. Rhythmic contractions at about 1.2 Hz of both Eustachian tubes, synchronous with the audible clicks, were observed, without any abnormal movement of the soft palate. The patient was able to voluntarily modulate the frequency of the audible clicks and could even transiently stop them (Video). Furthermore, when the patient was asked to open and close his hand at different speeds imposed by the examiner (averaging 1 Hz for the slow pace and 1.7 Hz for the fast pace), the pace of the audible clicks entrained to the imposed frequency. The same synchronous adaptation could be transiently elicited by setting an audible metronome rhythm at 0.75, 1.5 and 2.5 Hz, with progressive but transient matching of frequency with the metronome. This was best achieved at 1.5 Hz (Video).

**Video:** **Patient’s clinical examination.** To facilitate the reader/listener, a blue dot has been incorporated to the video each time there was a clicking sound. We recommend listening to the video at the highest available volume. 00:00 Baseline audible clicks from outside the right ear canal. 00:03 No visible movements of the right eardrum. 00:08 Right Eustachian tube visible movements (no sound). 00:18 Entrainable clicks with hand movements. 00:34 Volitional modification of ear clicks frequency. 00:54 Synchronization of ear clicks with metronome frequency.

Pure tone audiometry, tympanometry and stapedial reflexes were normal. Full brain MRI with thin cuts was normal. A trial with Clonazepam 0,5 mg bid for a few weeks didn’t change the symptomatology and was stopped by the patient.

## Discussion

Objective tinnitus can be caused by temporomandibular joint mechanical disorders, vascular abnormalities, patulous Eustachian tubes, middle-ear myoclonus (MEM), or associated with PT. Symptomatology can be distressful, requiring psychological support. Management options include medical treatments such as benzodiazepines and anticonvulsants, botulinum toxin injections and middle-ear muscles tendon section in selected cases [1].

Many muscles have been reported as being able to elicit tinnitus in the form of a clicking sound, including rhythmic contractions of the stapedial muscle (cranial nerve (CN) VII) or tensor tympani (CN V_3_) in MEM, and contraction of peritubal muscles (Figure). MEM refers to a tinnitus due to dysfunctional movement of either the tensor tympani or stapedius muscles [2,3,4,5]. The variety of sounds described in the setting of MEM goes well beyond clicking sounds (e.g., cracking, bobbling, blowing, whooshing etc…). MEM etiology seems to lump a number of different causes, from presumed “peripheral” causes such as associated with hemifacial spasm, to more typical central causes [2]. We would point out that the vast majority of patients with rhythmic clicking (and a presumed “central” cause) have concurrent palatal involvement. Rarely, clicks have also been described as originating from the base of the tongue [6].

**Figure:**
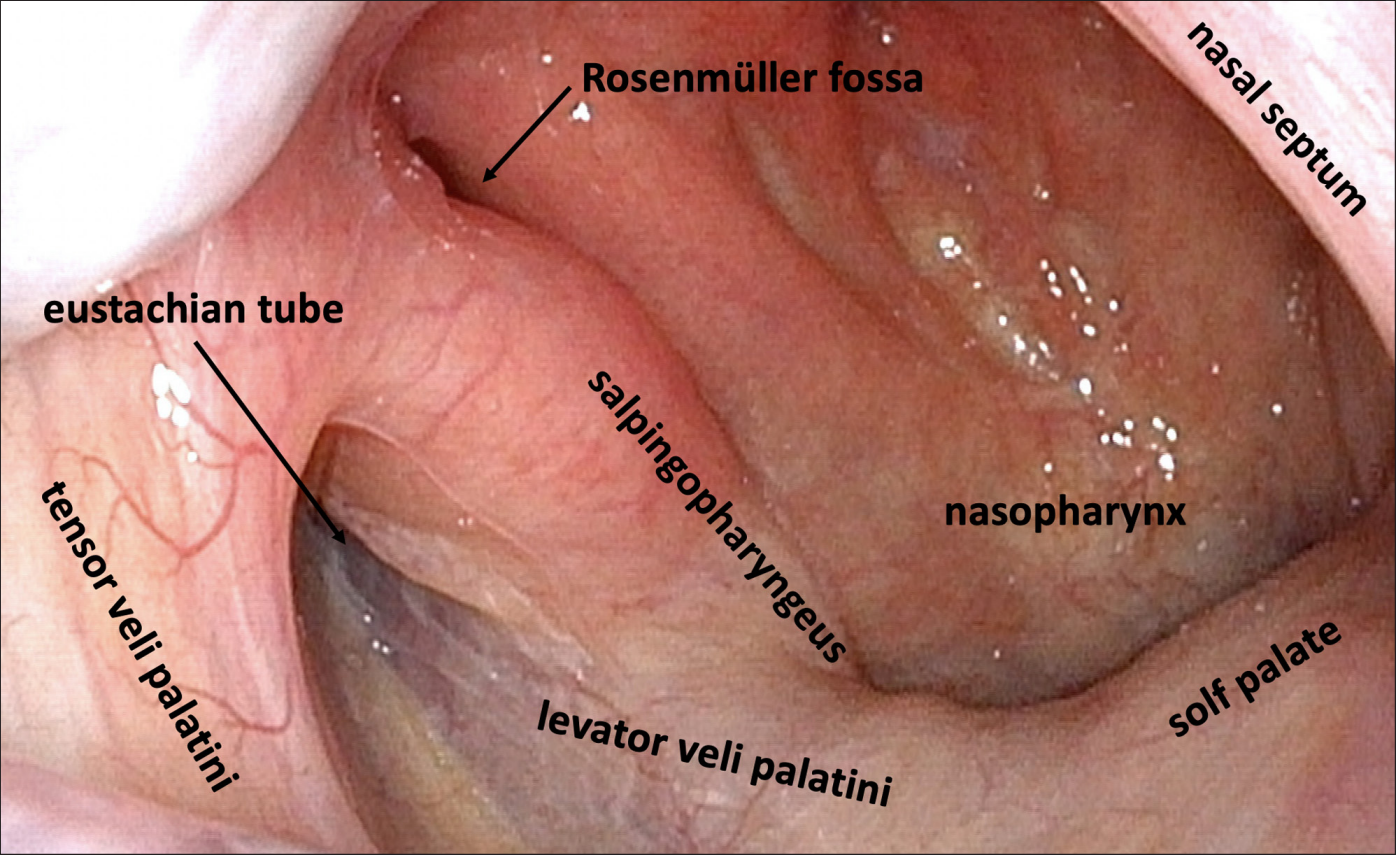
Right normal Eustachian tube by a healthy 32-year-old man with Eustachian and surrounding muscles legends (copyright belongs to François Voruz).

In case of ear clicks due to peritubal muscle contractions, changes in apposition of Eustachian tube walls by repetitive openings is believed to produce the sound [2]. These cases usually have associated PT due to contractions of either the tensor veli palatini (CN V_3_) in *essential* or *isolated* PT, or the levator veli palatini (CN IX and X) in the rare cases of *symptomatic* PT with ear clicks [7,8]. Another peritubal muscle, the salpingopharyngeus (CN X), can create tinnitus generally without moving the soft palate. Further support for this origin is a case report of a patient with tinnitus and PT who had previously failed botulinum toxin injections in the soft palate, whose tinnitus responded partially to botulinum toxin injection isolated to the salpingopharyngeus muscle [9]. This latter mechanism is believed to be causative in our case, due to the absence of visible palatal or eardrum movement.

By comparison with classical PT, the absence of both an MRI lesion and associated neurological signs would point towards an “*essential*” nosology in this isolated ear click case. Essential/isolated [10] (i.e. non-symptomatic) PT is divided into primary (no known etiology) and secondary forms, the latter being further sub-divided into functional or psychogenic PT where the movements are experienced as (i) involuntary, or lacking in self-agency, (ii) semi-volitional (e.g. tics) and (iii) voluntary/fully volitional (e.g. special skills) movements [10,11,12]. In a recent case report by Kern and Lang [11], the authors presented arguments for the possibility that some patients with “functional palatal tremor” may acquire the movement as a compensation for longstanding middle ear disease rather than having to invoke a “psychogenic” origin in all “functional” cases. Although a history of pre-existing middle ear infections was lacking, the present case fits best in most other respects with the “functional but non-psychogenic” classification, as proposed by Kern and Lang, with the lack of self-agency, including the lack of preceding urge, waxing and waning or other tics, absence of clear psychological contributing factors, and the presence of distractibility and entrainment. The awareness of partial volitional control is similar to other movement disorders (e.g., tremor in Parkinson’s disease, orolingual movements in tardive dyskinesia) but does not assist in classifying the movement further.
